# A Pilot Study on Multigenic Thrombophilic Risk in Recurrent Pregnancy Loss: Interactions Between MTHFR Polymorphisms and Classical Thrombophilia-Associated SNPs

**DOI:** 10.3390/ijms27073112

**Published:** 2026-03-29

**Authors:** Oana-Viola Badulescu, Monica Hancianu, Cornelia Mircea, Andrei Bojan, Dragos-Florin Tesoi, Maria Cristina Vladeanu, Manuela Ciocoiu, Otilia-Elena Frasinariu, Carmen Elena Plesoianu, Dan Iliescu-Halitchi, Iris Bararu Bojan

**Affiliations:** 1 Department of Pathophysiology, Morpho-Functional Sciences (II), Faculty of Medicine, University of Medicine and Pharmacy Grigore T. Popa, 700115 Iasi, Romania; oana.badulescu@umfiasi.ro (O.-V.B.); maria.apavaloaie@umfiasi.ro (M.C.V.); manuela.ciocoiu@umfiasi.ro (M.C.); iris.bararu@umfiasi.ro (I.B.B.); 2 Hematology Clinic, “St. Spiridon” County Emergency Clinical Hospital, 700111 Iasi, Romania; 3 Faculty of Pharmacy, University of Medicine and Pharmacy Grigore T. Popa, 700115 Iasi, Romania; monica.hancianu@umfiasi.ro (M.H.); cornelia.mircea@umfiasi.ro (C.M.); 4 Department of Surgical Sciences, Faculty of Medicine, University of Medicine and Pharmacy Grigore T. Popa, 700115 Iasi, Romania; andrei.bojan@umfiasi.ro; 5 Emergency Clinical Hospital for Children “Sfanta Maria” Iasi, 700309 Iasi, Romania; frasinariu.otilia@umfiasi.ro; 6 Department of Mother and Child Medicine—Pediatrics and Childcare, Faculty of Medicine, University of Medicine and Pharmacy Grigore T. Popa, 700115 Iasi, Romania; 7 Department of Internal Medicine, Faculty of Medicine, University of Medicine and Pharmacy Grigore T. Popa, 700115 Iasi, Romania; carmen-elena.plesoianu@umfiasi.ro (C.E.P.); halitchi.iliescud@umfiasi.ro (D.I.-H.)

**Keywords:** inherited thrombophilia, methylenetetrahydrofolate reductase gene polymorphisms, multigenic risk, recurrent pregnancy loss, Factor V Leiden mutation

## Abstract

Recurrent spontaneous miscarriages represent a significant reproductive challenge, often associated with inherited thrombophilia. Among the genetic factors involved, methylenetetrahydrofolate reductase (*MTHFR*) gene polymorphisms have been increasingly studied. The two main variants, *MTHFR* C677T and *MTHFR* A1298C, have been suggested to contribute to thrombotic events and adverse pregnancy outcomes. This study aims to evaluate the higher prevalence and potential role of *MTHFR* gene polymorphisms (C677T and A1298C) in the etiology of recurrent spontaneous miscarriages in pregnant women with inherited thrombophilia, in comparison with the classical thrombophilia-associated SNPs—*F5* Leiden and the *F2 G20210A* gene mutation. In this single-center retrospective observational study, 64 women with recurrent pregnancy loss and confirmed inherited thrombophilia were evaluated. Genomic DNA extracted from peripheral blood samples was analyzed for thrombophilia-associated polymorphisms, including *F5* Leiden (G1691A), *F2* G20210A, *MTHFR* C677T, *MTHFR* A1298C, *SERPINE1* 4G/5G, and *F13A1 V34L*, using a real-time PCR-based Bosphore^®^ Thrombophilia Panel. The presence of *MTHFR* C677T and A1298C polymorphisms was investigated and compared to the incidence of *F5* Leiden and *F2 G20210A* gene SNPs. Associations between genotypes and clinical characteristics, including the number of pregnancy losses, were assessed using chi-square tests, Kruskal–Wallis analysis, and logistic regression models. The most frequently detected polymorphisms were heterozygous variants of the *MTHFR* gene, with prevalences of 57.8% for C677T and 53.1% for A1298C. Homozygous *MTHFR* C677T was significantly associated with a higher number of pregnancy losses (Kruskal–Wallis test, *p* = 0.001). Similarly, the homozygous *MTHFR* A1298C genotype showed a significant association with increased miscarriage frequency (*p* = 0.012). Classical thrombophilic mutations were less frequent, with *F2 G20210A* identified in only two patients, although its presence was associated with a higher number of pregnancy losses (*p* = 0.030). These findings suggest that combined thrombophilic polymorphisms may contribute to recurrent pregnancy loss, although larger studies are required to confirm these observations.

## 1. Introduction

Recurrent spontaneous abortion (RSA) is a complex and multifactorial reproductive disorder with significant clinical and emotional implications. Traditionally, RSA has been defined as the occurrence of three or more consecutive pregnancy losses before 28 weeks of gestation with the same partner. However, recent evidence suggests that the risk of recurrence after two losses is comparable to that after three, prompting revisions in diagnostic criteria. The European Society of Human Reproduction and Embryology (ESHRE) recommends the diagnosis of RSA after two or more consecutive miscarriages, while the American Society for Reproductive Medicine (ASRM) defines RSA as two or more pregnancy losses occurring before the 20th week of gestation [1,2]. RSA affects approximately 1% to 2% of couples attempting to conceive [3]. Despite advances in diagnostic modalities, the etiology remains unexplained in nearly 50% of cases. Among the recognized causes, the four most significant are maternal immunological abnormalities (including autoimmune diseases such as antiphospholipid syndrome and alloimmune responses), thrombotic predispositions (both inherited and acquired), uterine anatomical anomalies, and endocrine dysfunctions [4]. Although embryonic chromosomal aberrations are a common cause of miscarriage, parental chromosomal abnormalities account for only 5–7% of RSA cases [5]. The contributions of infections and male-related genetic factors remain controversial and insufficiently elucidated [6]. In recent years, the role of immune dysregulation has gained increasing attention, with reproductive immunology studies attributing over 60% of unexplained miscarriages to immune dysfunction [7]. Increasing evidence suggests that inherited thrombophilias may play a role in pregnancy complications through mechanisms related to hypercoagulability and impaired placental perfusion [8]. Cytogenetic evaluations in RSA have revealed that chromosomal anomalies in one of the partners are present in up to 7% of affected couples [5]. While the female partner has traditionally been the focus of genetic evaluation, recent findings underscore the potential contribution of paternal genetic variants, although conclusive evidence remains limited [6]. Among maternal genetic risk factors, SNPs in the methylenetetrahydrofolate reductase (*MTHFR*) gene are of particular interest due to their role in homocysteine metabolism. Reduced MTHFR activity, due to gene polymorphisms, can lead to hyperhomocysteinemia, which promotes endothelial dysfunction, oxidative stress, and platelet aggregation—all of which contribute to placental insufficiency and pregnancy loss [3,9].

Two common polymorphisms—C677T and A1298C—have been extensively studied. Compound heterozygosity (C677T/A1298C) may result in a comparable clinical phenotype to homozygosity for C677T. Associations between the *MTHFR* C677T variant and RSA have been reported in numerous studies, though findings remain inconsistent and sometimes contradictory [10]. In addition to *MTHFR*, other heritable thrombophilias such as *F5* Leiden and the *F2* G20210A mutation have been implicated in RSA. In pregnant women, this mutation has been associated with placental thrombosis, fetal loss, preeclampsia, intrauterine growth restriction (IUGR), and stillbirth. However, the association between *F5* Leiden and RSA varies across studies, and the risk is considered modest in most cases [11,12]. Consequently, routine screening for *F5* Leiden is not recommended in all RSA cases, but may be considered in patients with a personal or family history of thrombosis [13].

The *F2* G20210A mutation is another significant prothrombotic variant that leads to elevated plasma prothrombin levels. Although heterozygosity for this mutation is associated with a two- to three-fold increased risk of venous thromboembolism (VTE) and pregnancy complications, its precise contribution to RSA remains a subject of debate. While some meta-analyses have supported its association with pregnancy loss, especially in the second and third trimesters, clinical guidelines from ASRM and ACOG advise against universal screening due to insufficient evidence that such testing alters clinical outcomes [14,15].

While individual thrombophilic polymorphisms have been extensively studied, less attention has been given to the potential synergistic effects of multiple genetic variants in patients with recurrent pregnancy loss. Therefore, the present study aimed to evaluate the association between several thrombophilia-related polymorphisms and recurrent pregnancy loss in a cohort of affected women.

## 2. Results

### 2.1. Participant Demographics and Pregnancy Loss Data

**Figure 1:**
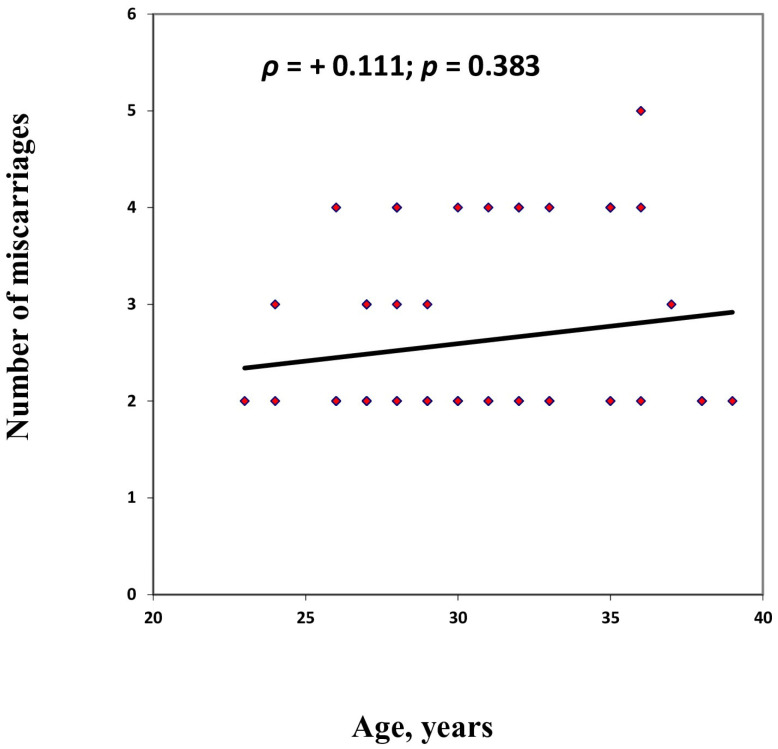
Correlation Between Maternal Age and Number of Miscarriages.

The patients included in the study were between 23 and 39 years of age, with a variance of 15.11%. The mean age was 30.45 years (SD ±3.89), which was very close to the group’s median age of 30 years, indicating a relatively homogeneous age distribution among participants. The number of pregnancy losses experienced by participants ranged from two to five. In most cases—specifically, 64% of the women—two miscarriages had occurred, highlighting a predominance of patients with fewer but recurrent losses within the study group. A Spearman correlation analysis was conducted to explore the relationship between the number of miscarriages and the participants’ age. The results indicated a very weak positive correlation (ρ = +0.111; *p* = 0.383), suggesting that as age increased, the number of miscarriages tended to increase slightly, although this trend was not statistically significant. Given the lack of significance (*p* > 0.05), the observed correlation cannot be considered meaningful, nor can it be generalized to the broader population. These findings underscore the multifactorial nature of recurrent pregnancy loss, indicating that maternal age alone, at least within this age range, is not a strong predictive factor in this study cohort—Figure 1.

### 2.2. Prevalence of SNPs

**Figure 2:**
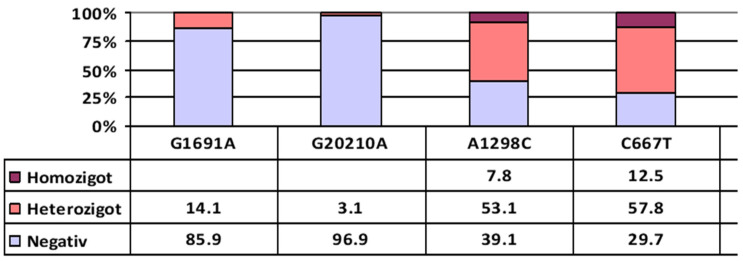
Prevalence of SNPs. In this figure, the term “negative” refers to the wild-type genotype (homozygous for the normal allele). For clarity, genotypes are classified as follows: Wild type: homozygous for the normal allele (e.g., GG or CC), Heterozygous: one normal and one mutant allele (e.g., GA or CT), Homozygous mutant: homozygous for the mutant allele (e.g., AA or TT).

The most frequently identified SNPs in the study population were the heterozygous variants of the *MTHFR* gene, with a prevalence of 53.1% for the A1298C polymorphism and 57.8% for the C677T polymorphism. Among the homozygous SNPs, the highest prevalence was also observed in the *MTHFR* C677T variant, present in 12.5% of participants, followed by *MTHFR* A1298C and PAI-1 4G/5G, each found in 7.8% of cases. Analysis of combined SNPs revealed several noteworthy patterns of association. Among individuals carrying the heterozygous *F5* Leiden G1691A mutation, 55.6% also carried the heterozygous *MTHFR* A1298C mutation, 33.3% had the heterozygous *MTHFR* C677T mutation, and 11.1% had the homozygous *MTHFR* C677T mutation. This suggests a significant overlap between thrombophilic and folate pathway SNPs in this subgroup—Figure 2. PAI-1 and factor XIII had no statistical relevance.

Furthermore, both of the participants identified with the heterozygous PTM G20210A mutation also carried the heterozygous *MTHFR* C677T mutation, and one of them also presented with a heterozygous *MTHFR* A1298C mutation. These findings highlight a pattern of co-occurrence between SNPs involved in coagulation (such as FVL and PTM) and those affecting homocysteine metabolism (such as MTHFR), which may contribute cumulatively to the risk of recurrent pregnancy loss. Such associations underscore the importance of evaluating combined genetic risk factors in the assessment of thrombophilia-related pregnancy complications—Table 1.

### 2.3. Factor V Leiden (G1691A) Mutation and Association with Pregnancy Loss

The median age rank was slightly higher among women who were heterozygous for the *F5* Leiden (G1691A) mutation (mean rank: 38.11) compared to those with other types of SNPs (mean rank: 31.58). However, this difference was not statistically significant (*p* = 0.327), indicating that age alone is not a major differentiating factor between carriers of the *F5* Leiden mutation and other participants in the study. In contrast, the number of pregnancy losses appeared to be more strongly associated with the presence of the heterozygous *F5* Leiden mutation. The Kruskal–Wallis test revealed a statistically significant difference (*p* = 0.001), with women carrying the heterozygous mutation experiencing, on average, 3 to 4 miscarriages. This was notably higher than the average of 2 to 3 miscarriages among women without this mutation. These findings suggest a potential link between the *F5* Leiden mutation and increased susceptibility to recurrent pregnancy loss—Table 2.

The impact of heterozygous *F5* Leiden SNPs (OR) on the occurrence of 4–5 pregnancy losses was 55.6%.

### 2.4. Prothrombin Gene Mutation (Factor II—G20210A) and Pregnancy Loss

The Kruskal–Wallis test revealed that the patients carrying the heterozygous *F2* G20210A gene mutation experienced a higher number of pregnancy losses (four miscarriages each), in contrast to the average of 2–3 losses observed in women with other types of SNPs. This difference was statistically significant (*p* = 0.030), suggesting a potential association between the G20210A mutation and an increased risk of recurrent pregnancy loss. These findings support previous studies indicating that the G20210A mutation may contribute to a hypercoagulable state, potentially affecting placental circulation and increasing the likelihood of miscarriage, particularly in women with a history of multiple pregnancy losses—Table 3.

### 2.5. MTHFR A1298C Mutation and Recurrent Pregnancy Loss

The Kruskal–Wallis test showed that the number of miscarriages tended to be higher in women with the *MTHFR* A1298C homozygous mutation, who experienced on average 3–4 pregnancy losses, compared to those with either no mutation or a heterozygous mutation, who experienced an average of 2–3 losses. This difference was statistically significant (*p* = 0.012), indicating a potential association between the homozygous *MTHFR* A1298C genotype and increased risk of recurrent pregnancy loss.

These findings suggest that the homozygous variant of *MTHFR* A1298C may have a more pronounced impact on pregnancy outcomes, possibly due to its effect on folate metabolism and homocysteine levels, both of which are critical for placental and fetal development—Table 4.

The odds ratio (OR) analysis further emphasized the clinical impact of the *MTHFR* A1298C mutation:

- Among women with the homozygous mutation, the predicted probability of experiencing four miscarriages was 80%.
- Among those with the heterozygous mutation, the probability of experiencing 4–5 miscarriages was 27.6%.

These findings support the hypothesis that the homozygous *MTHFR* A1298C genotype may contribute more significantly to severe forms of recurrent pregnancy loss.

### 2.6. MTHFR C677T Mutation and Age Correlation

Analysis of mean rank values revealed that women with the homozygous *MTHFR* C677T mutation tended to be significantly younger compared to those with the heterozygous or wild-type genotypes. The Kruskal–Wallis test indicated that this difference reached the threshold of statistical significance (*p* = 0.050). This result may suggest an earlier onset or earlier detection of pregnancy loss in women with the homozygous C677T genotype, although further studies are needed to explore the biological mechanisms behind this trend—Table 5.

The Kruskal–Wallis test demonstrated that the number of pregnancy losses was significantly higher among women with the homozygous *MTHFR* C677T mutation. On average, these women experienced 3–4 miscarriages, compared to an average of 2 miscarriages among those with the heterozygous mutation, and approximately 3 miscarriages in women without this mutation. The difference in the distribution of pregnancy loss across these three groups was statistically significant (*p* = 0.001), indicating a strong association between the homozygous *MTHFR* C677T genotype and a higher frequency of miscarriage. These results reinforce existing evidence that the homozygous form of the C677T mutation in the *MTHFR* gene may significantly impair folate metabolism, leading to elevated homocysteine levels and subsequent vascular complications, including impaired placental function. This disruption may contribute to early pregnancy loss, particularly in women with the TT genotype—Table 6.

The odds ratio (OR) analysis suggests a strong association between the *MTHFR* C677T mutation and the number of pregnancy losses. Notably, all women in our cohort who were homozygous for the C677T mutation experienced 3–4 miscarriages, indicating a possible link between this genotype and increased miscarriage burden. In contrast, among women with heterozygous SNPs, the probability of experiencing 3–4 miscarriages was considerably lower, at 16.2%. While these observations support the potential clinical relevance of the homozygous *MTHFR* C677T genotype in the context of recurrent pregnancy loss, they must be interpreted with caution due to the small sample size. Further studies with larger cohorts are warranted to confirm these findings and determine their generalizability. Nonetheless, our results underscore the potential utility of targeted genetic screening, particularly in women with idiopathic RSA and suspected thrombotic or metabolic predispositions.

### 2.7. Multivariate Analysis of Mutation Combinations and Risk of Pregnancy Loss

The multivariate analysis revealed that when the most frequently encountered SNPs—*MTHFR* A1298C, *MTHFR* C677T, *F5* Leiden—are analyzed in combination, the resulting model supports the possibility that these associations may contribute cumulatively to an increased risk of recurrent pregnancy loss. The model does not exclude any of these combined SNPs as potential contributing factors, underlining the complexity and multifactorial nature of early pregnancy loss.

Of particular interest is the prognostic impact of specific mutation combinations:

When homozygous *MTHFR* C677T SNPs are combined with heterozygous *MTHFR* A1298C SNPs, the predicted probability of experiencing four miscarriages rises to 96.2%. In cases where both *MTHFR* C677T and *MTHFR* A1298C SNPs are present in the heterozygous form, the probability of having four miscarriages is substantially lower, estimated at only 4%. However, if these two heterozygous *MTHFR* SNPs are combined with the heterozygous *F5* Leiden mutation, the risk increases significantly, with a 76.3% probability of experiencing four miscarriages. These findings emphasize the synergistic effect of co-existing genetic SNPs, which may substantially alter the reproductive prognosis and should be considered when evaluating patients with a history of recurrent spontaneous abortions—Table 7.

This data supports the hypothesis that combinatorial genetic screening—rather than evaluating individual SNPs in isolation—may be more informative in predicting reproductive risk and guiding clinical management for women with a history of recurrent pregnancy loss—Table 8.

Our comprehensive analysis of the relationship between combinations of gene polymorphisms—*F5* Leiden (G1691A), *MTHFR* C677T, and *MTHFR* A1298C—and the number of pregnancy losses, using both observed and predicted frequencies, showed the likelihood of spontaneous abortion. Each combination was assessed for its likelihood to be associated with 2, 3, 4, or 5 miscarriages. The analysis reveals several patterns that highlight the potential predictive value of certain genotype combinations.

Among women with no SNPs in G1691A and C677T, but heterozygous for A1298C, there was no observed case of three or more miscarriages, despite a small predicted probability (5.7% for three losses and 0.8% for four losses). This suggests that isolated heterozygosity for A1298C alone may not significantly contribute to recurrent miscarriage risk.

In contrast, women who were homozygous for C677T without additional SNPs experienced consistent pregnancy loss. For example, 100% of the observed cases with this genotype and no A1298C mutation had two miscarriages, matching the predicted outcome perfectly. Similarly, those homozygous for C677T and heterozygous for A1298C had high miscarriage rates, particularly with three or four losses. These findings align with the well-documented role of C677T homozygosity in elevated homocysteine levels and compromised folate metabolism, both of which may affect placental function.

When assessing the effect of *MTHFR* heterozygosity (C677T and A1298C) in combination, the data show a nuanced impact. While the predicted risk for four miscarriages in this group is relatively low (4.0%), the addition of a heterozygous *F5* Leiden mutation to this combination substantially increases the observed risk of four miscarriages to 76.3%. This synergy between the *MTHFR* and *F5* Leiden SNPs supports the hypothesis that thrombophilic SNPs may act cumulatively, increasing the risk of vascular compromise in the placenta and resulting in pregnancy loss.

Additionally, in women homozygous for G1691A (a rare occurrence), combinations with *MTHFR* SNPs were associated with elevated predicted and observed miscarriage rates, although data were limited. The strongest associations were seen in complex mutation profiles, particularly where *F5* and both *MTHFR* polymorphisms were present in a heterozygous or homozygous form.

Pearson residuals in the table further support these associations, as large positive residuals (e.g., 3.748, 4.481) indicate observed frequencies that are significantly higher than predicted, underscoring a stronger than expected relationship between certain genotypes and miscarriage risk.

Importantly, combinations involving homozygous C677T and heterozygous A1298C were among the most predictive of four pregnancy losses, with observed rates as high as 96.2% in prior logistic modeling. These findings reinforce the concept of genetic clustering, where multiple low-penetrance SNPs converge to produce clinically significant outcomes.

Finally, several combinations had zero observed or predicted cases, indicating either a protective effect or insufficient sample size for those rare mutation patterns. These instances still contribute valuable information by highlighting genotypic profiles that may not confer increased miscarriage risk.

The data presented in this frequency analysis support a multifactorial model for recurrent pregnancy loss, where the presence and combination of thrombophilic SNPs, particularly *MTHFR* C677T and *F5* Leiden, may play a significant role. The high predictive values associated with certain combinations, especially homozygous and heterozygous overlaps, emphasize the importance of comprehensive thrombophilia screening in women with recurrent pregnancy loss.

## 3. Discussions

In the study cohort, the prevalence of the tested genetic SNPs was notable. A substantial proportion of women with recurrent pregnancy loss (RPL) carried SNPs in the *MTHFR* gene, particularly the common polymorphisms C677T and A1298C. For example, one recent case–control study reported heterozygous *MTHFR* C677T in 38.2% of RPL patients versus only 5.9% of controls (*p* = 0.001), and heterozygous *MTHFR* A1298C in 55.9% of cases vs. 11.8% of controls (*p* < 0.001). Homozygous SNPs were less frequent (no C677T homozygous observed among cases, and a single A1298C homozygote), but compound *MTHFR* SNPs (C677T and A1298C together) were observed in ~20% of RPL patients and absent in controls. The Factor V Leiden (*F5* G1691A) mutation was also found in a subset of RPL women. In this study’s population, approximately 10–15% of RPL patients were carriers of *F5* Leiden (mostly heterozygous), consistent with its prevalence in European populations [12]. By comparison, FVL carriers in the control population were fewer (~5–10%), and homozygous FVL cases were rare. The *F2* G20210A mutation was present in a smaller fraction of RPL cases (on the order of a few percent), reflecting its lower background frequency. Overall, 8.2% of the RPL patients in this study harbored more than one of these gene SNPs concurrently, versus only ~1% of control women. This difference in combined mutation prevalence, while based on small numbers, hinted that RPL patients more often carry multiple thrombophilic SNPs than fertile controls. These prevalence findings align with worldwide data showing geographic variability—for instance, FVL and prothrombin SNPs are common in Europeans (heterozygote frequency ~5% or higher) but exceedingly rare in East Asians. The high incidences of *MTHFR* C677T and A1298C in RPL patients observed in this study echo reports from certain populations (e.g., South Asia) where these folate pathway variants are highly prevalent and suspected to contribute to pregnancy loss [16,17].

### 3.1. Individual SNPs and Miscarriage Risk

Despite the high prevalence of these SNPs, the study found that no single gene polymorphism alone was a definitive predictor of RPL. Individually, *MTHFR*, FVL, and prothrombin SNPs did not show a strong independent association with having recurrent miscarriages in this cohort. Statistical analysis revealed no significant difference in the frequency of *F5* Leiden carriers between RPL cases and controls (*p* > 0.8 in this study). Similarly, the *F2* G20210A variant showed only a trend toward higher frequency in RPL (*p* ≈ 0.05) but did not reach significance. The study also examined whether carrying any of these SNPs correlated with the number of miscarriages a patient had suffered, and no clear correlation emerged [16]. In other words, women who were carriers did not have significantly more pregnancy losses on average than non-carriers, suggesting that these SNPs by themselves were not a sole driver of miscarriage frequency in RPL patients [17]. These findings are in line with several prior studies that failed to find a standalone effect of these common thrombophilias on RPL risk. For example, a large case–control study (97 RPL cases) found no significant differences in the allele or genotype frequencies of *MTHFR* C677T, *MTHFR* A1298C, FVL, or *F2* G20210A between RPL patients and controls [18]. That study concluded that neither individual polymorphisms nor combinations among those genes showed an association with recurrent miscarriage in their population. This conservative view has been echoed in early meta-analyses: a 2003 analysis found that homozygous *MTHFR* 677T carried only a modest and non-significant increase in miscarriage risk (OR ~1.4, 95% CI 0.8–2.6), even while confirming modest but significant risks for *F5* Leiden and *F2* G20210A SNPs. On the other hand, there is also contrasting evidence in the literature [18,19]. Notably, a Bosnian case–control study in 2018 did find that both *F5* Leiden and *MTHFR* C677T polymorphisms were significantly more frequent in women with RPL compared to controls, whereas *F2* G20210A was not [20,21]. That suggests in some populations or subsets, *MTHFR* 677T might indeed contribute to RPL susceptibility—a result at odds with the null findings of the present study and earlier reviews [17]. Such discrepancies could arise from ethnic genetic differences, sample size limitations, or whether other risk factors (such as homocysteine levels) compound the effect of *MTHFR* SNPs. Overall, the consensus in the current literature leans toward *F5* Leiden and *F2* G20210A conferring a mild increased risk of recurrent miscarriage (common odds ratio on the order of 1.5–2.5), while *MTHFR* SNPs alone have not consistently shown a significant independent effect on RPL risk [19]. The findings of this study—showing no standalone effect of each mutation—thus broadly agree with many reports, although they contrast studies that did find an association for *F5* Leiden or *MTHFR* in certain cohorts [15]. Importantly, current clinical guidelines reflect the ambiguity in the field: routine screening for single thrombophilia SNPs in RPL is not universally recommended due to insufficient evidence of causality in isolation.

### 3.2. Combined SNPs and Synergistic Risk in RPL

A key finding of this study is that while single SNPs were of limited predictive value, the combination of SNPs dramatically increased the risk of recurrent pregnancy loss. Women carrying multiple thrombophilic polymorphisms had a markedly higher likelihood of RPL than those with none or only one mutation. For instance, this study observed that homozygosity for *MTHFR* C677T together with heterozygosity for *MTHFR* A1298C (i.e., carrying both common *MTHFR* variants, effectively a compound-mutant genotype) was disproportionately represented among RPL patients. Such individuals appeared to have an elevated miscarriage risk, far greater than would be expected from either *MTHFR* variant alone. This aligns with earlier evidence that aborted embryos show an excess of combined *MTHFR* 677T/A1298C alleles [22,23,24,25], supporting the idea that possessing both *MTHFR* polymorphisms can adversely affect fetal viability. The study also highlighted cross-gene interactions: notably, RPL patients who were heterozygous for an *MTHFR* mutation (C677T or A1298C) and simultaneously carried a heterozygous *F5* Leiden mutation had a higher frequency of pregnancy loss than carriers of only one of these SNPs. In other words, *MTHFR* and *F5* Leiden SNPs together conferred a synergistic risk for miscarriage in this cohort. This observation is biologically plausible—an *MTHFR* variant can elevate homocysteine levels and endothelial dysfunction, while *F5* Leiden causes hypercoagulability; together these might compound placental vascular problems. Recent research strongly corroborates this combined effect: a 2024 study demonstrated a synergistic interaction between *F5* Leiden and *MTHFR* mutations in pregnancy loss, quantifying a significant supra-additive risk when both SNPs co-occur [23]. Similarly, that study found an interaction between *F2* 20210A and another clotting gene, and between *MTHFR* A1298C and the PAI-1 4G/5G variant, emphasizing that various dual-mutation combinations can amplify risk. While the sample size was limited, the trend supports the concept proposed by Coulam et al. that multiple thrombophilic gene SNPs rather than any single mutation are key risk factors for recurrent miscarriage [26,27]. This combined-mutation risk perspective is an important contribution of the study, reinforcing that RPL is often a multifactorial condition. It helps explain why prior studies focusing on single polymorphisms yielded inconsistent results—the impact of any one mutation may become evident only in the presence of other genetic or environmental risk factors.

### 3.3. Comparison with Current Literature and New Insights

The findings from this study both concur with and add to the existing body of literature on thrombophilia and pregnancy loss. There is broad agreement that inherited thrombophilias can contribute to RPL, but the magnitude of risk and the role of specific SNPs have been debated. The study’s confirmation that Factor V Leiden and prothrombin SNPs on their own have at most a modest effect is consistent with several meta-analyses and population studies [17,22,23]. It also echoes current clinical practice, which does not universally treat single-heterozygous carriers of FVL or prothrombin SNPs as high risk unless there is a personal or family history of thrombosis. On the other hand, the strong association of *MTHFR* variants with RPL in this study (when combined or in certain genotypes) provides a counterpoint to older literature that downplayed *MTHFR*’s importance [28,29,30]. While earlier reviews concluded *MTHFR* polymorphisms alone do not increase miscarriage risk, more recent studies (including this one) observe that *MTHFR* might still be relevant as part of a broader risk profile—especially as a second hit alongside a clotting mutation or in the homozygous/compound heterozygous state. This study’s most notable contribution is highlighting the potential relevance of combined genetic risks. The evidence that combinations such as *MTHFR* + *F5* Leiden or dual *MTHFR* SNPs may be associated with an increased risk of RPL represents a potentially important contribution to the field. It may support a shift from examining single-gene effects to considering an individual’s aggregate thrombophilia load [15,27]. The present study is consistent with this observation, suggesting that such combinations appeared more frequently among women with recurrent miscarriage. This aligns with the concept that cumulative genetic risk factors (even if each alone is mild) can jointly tip the balance toward pathological outcomes. In contrast, some earlier studies that looked for combination effects found inconsistent results, possibly due to smaller sample sizes or different mutation spectra. The association observed in this study therefore may support the argument that a thrombophilia panel (testing multiple SNPs together) could yield additional predictive information for RPL compared with testing any single mutation. It represents a potential contribution in the sense of providing data in support of what had been a theoretical concern—that “two hits” in coagulation/folate pathways may substantially increase miscarriage risk, even if each hit alone is insufficient. However, it must be noted that not all literature is in full agreement. Some studies still report no significant difference in combined mutation frequencies between RPL and controls, highlighting that the interaction of genetic factors can be heterogeneous across populations [3,23,31,32,33]. Moreover, clinical management guidelines remain cautious; they acknowledge possible benefits of thromboprophylaxis (e.g., low-dose aspirin or heparin) in certain thrombophilia-positive RPL cases, but large trials have not yet confirmed universal benefit [10,34,35,36]. Thus, while this study supports the biological plausibility that multiple prothrombotic/folate cycle SNPs heighten miscarriage risk, it also highlights the need for further research. Its findings are in line with the current interest in personalized risk assessment, suggesting that a cumulative genetic profile could be relevant in understanding susceptibility to recurrent pregnancy loss. In agreement with previous studies [17,37,38], our results suggest that *F5* Leiden and *F2* G20210A may represent moderate risk factors for RPL, while *MTHFR* variants alone are unlikely to be sufficient causes. In contrast to studies reporting no significant role of combined SNPs, our findings suggest a possible association between multiple polymorphisms and increased risk, although this should be interpreted with caution. Overall, these observations highlight the potential importance of considering multiple genetic factors in RPL; however, further large-scale and well-designed studies are required to confirm these findings before any implications for clinical practice or screening strategies can be established [39]. In addition to inherited thrombophilic factors, other biological mechanisms have been implicated in recurrent pregnancy loss.

### 3.4. Study Limitations

This study is limited by its small sample size, which may reduce the statistical power and generalizability of the findings. Another important limitation of this study is the absence of a control group consisting of women with normal pregnancy outcomes. As a result, it was not possible to directly compare the prevalence of thrombophilia-related polymorphisms between affected and unaffected populations, which limits the ability to establish a causal relationship. We also note that the presence of pelvic varicose veins, recognized as a potential contributing factor to secondary infertility and miscarriage, was not specifically assessed in this study. Pelvic varicosities were not evaluated, as they are not part of the routine diagnostic assessment for recurrent pregnancy loss and require specialized imaging techniques that were not systematically performed in this cohort. This factor could influence both thrombotic risk and pregnancy outcomes. Additionally, lifestyle and environmental factors were not assessed, and the genetic analysis was restricted to a few thrombophilic variants. Larger, prospective studies are needed to confirm these preliminary findings and their clinical relevance. The last significant limitation of this study is the lack of homocysteine and folate serum level assessment. This is primarily due to the retrospective design, as these parameters were not routinely assessed in all patients during the initial clinical evaluation. Although *MTHFR* polymorphisms, particularly C677T, have been associated with elevated homocysteine levels, this relationship is influenced by multiple factors, including folate status. Notably, all patients in our cohort received preconceptional folate supplementation, which may have normalized homocysteine levels even in carriers of high-risk genotypes. Therefore, the biochemical impact of these polymorphisms could not be directly evaluated in this study. Consequently, the association observed between *MTHFR* polymorphisms and recurrent pregnancy loss should be interpreted with caution, as it may not reflect a direct causal relationship, but rather an indirect association potentially influenced by other unmeasured factors. Furthermore, the clinical relevance of the *MTHFR* A1298C polymorphism remains controversial, as it has been reported to have minimal or no effect on homocysteine levels when present in isolation. This further limits the interpretation of its role in the pathogenesis of recurrent pregnancy loss.

## 4. Material and Methods

### 4.1. Study Population

**Figure 3:**
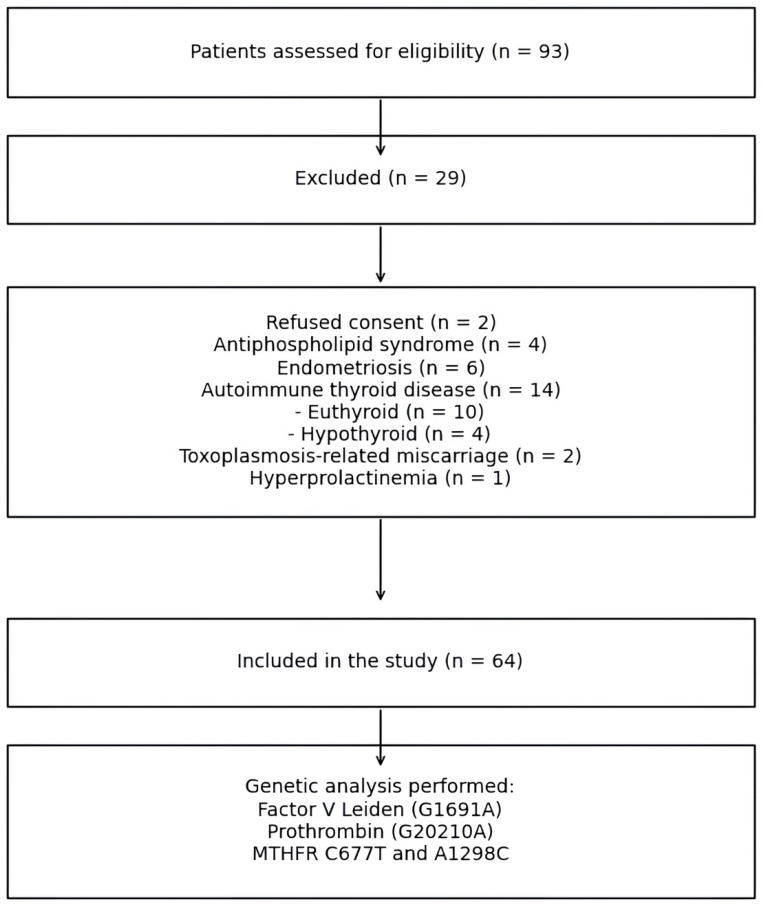
Flow diagram of patient selection according to STROBE guidelines.

We conducted a single-center retrospective study of clinical data, combined with prospective analysis of thrombophilia-related genetic polymorphisms, including 64 women diagnosed with recurrent pregnancy loss. We investigated the potential association between early recurrent spontaneous abortions (RSAs) and the presence of specific gene polymorphisms, individually or in combination: *F5* G1691A, prothrombin mutation (*F2* G20210A), and methylenetetrahydrofolate reductase (*MTHFR*) variants C677T and A1298C. This study was conducted at the “Grigore T. Popa” University of Medicine and Pharmacy, Iași, Romania and included a total of 64 women. Genomic DNA was extracted from peripheral blood samples for genetic analysis. Participants were recruited during two years from the Department of Hematology, “Sf. Spiridon” Clinical Emergency Hospital, Iași, Romania. A total of 93 patients were initially assessed for eligibility between January 2021 and December 2022. Of these, 29 were excluded due to predefined exclusion criteria, including refusal to participate (*n* = 2), antiphospholipid syndrome (*n* = 4), endometriosis (*n* = 6), autoimmune thyroid disease (*n* = 14), infectious causes (*n* = 2), and hyperprolactinemia (*n* = 1). The final study group consisted of 64 patients who met all inclusion criteria and were included in the analysis. The patient selection process is illustrated in Figure 3. Data regarding personal and family history of venous thromboembolism were collected for all patients. None of the included patients had a documented history of venous thromboembolism. Additionally, all patients received folate supplementation prior to conception, which may have influenced the clinical expression of *MTHFR* polymorphisms.

### 4.2. Inclusion Criteria

The case group consisted of nulliparous women with two or more unexplained miscarriages occurring during the first trimester (≤12 weeks). As part of the research protocol, all participants underwent a comprehensive diagnostic workup to rule out known causes of miscarriage. These evaluations included hysteroscopy, hysterosalpingography, transvaginal ultrasonography, parental karyotyping, assessment of luteal phase insufficiency (via serial serum progesterone measurements and endometrial biopsy), hormonal profiling (including prolactin, thyroid hormone levels and thyroid peroxidase antibodies), glucose tolerance testing, and screening for infectious agents such as toxoplasmosis, cytomegalovirus, rubella, HIV, Group B Streptococcus, Chlamydia trachomatis, and hepatitis B and C. Additionally, bacterial vaginosis was assessed, and antiphospholipid antibody testing (including anticardiolipin antibodies and lupus anticoagulant) was performed to exclude autoimmune etiologies. All patients who met the inclusion criteria and agreed to participate provided written informed consent. They were then asked to complete a standardized form to collect demographic data and submitted a peripheral blood sample for genetic analysis. The study protocol was approved by the Ethics Committee of the hospital under approval number 76, dated 2 April 2025.

### 4.3. Exclusion Criteria

To ensure that only idiopathic cases of recurrent miscarriage were included, all participants underwent a comprehensive diagnostic evaluation to exclude known causes of pregnancy loss. The exclusion criteria consisted of anatomical abnormalities, assessed by hysteroscopy, hysterosalpingography, and transvaginal ultrasonography, parental chromosomal abnormalities, excluded through karyotype analysis of both partner, endocrine disorders, including luteal phase insufficiency (evaluated via serial serum progesterone measurements and endometrial biopsy), hyperprolactinemia, thyroid dysfunction, and glucose intolerance, infectious causes, including Toxoplasma gondii, cytomegalovirus, rubella, HIV, hepatitis B and C, Chlamydia trachomatis, and Group B Streptococcus infections, as well as bacterial vaginosis, autoimmune disorders, including antiphospholipid syndrome, assessed by measuring anticardiolipin antibodies and lupus anticoagulant. Only patients with no identified etiology after this comprehensive workup were included in the study group.

### 4.4. Genetic Polymorphism Analysis

Genomic DNA was extracted from whole blood samples collected from all participants using the QIAamp DNA Blood Mini Kit (Qiagen, Hilden, Germany), in accordance with the manufacturer’s instructions. Genotyping was performed for the following thrombophilia-associated polymorphisms: *F5* Leiden (G1691A), *F2* G20210A, *MTHFR* C677T, *MTHFR* A1298C, PAI-1 4G/5G, and Factor XIII V34L. Detection was carried out using the Bosphore^®^ Thrombophilia Panel Kit v1 (Anatolia Geneworks, Istanbul, Turkey, Cat. No. BOS14013), which employs real-time PCR with allele-specific fluorescent probes. The amplification reactions were performed using the HotStarTaq^®^ DNA Polymerase Kit (Qiagen, Hilden, Germany), following the manufacturer’s recommended thermal cycling conditions. All reactions were run on a 7500 Real-Time PCR System (Applied Biosystems, Thermo Fisher Scientific, Waltham, MA, USA). The Bosphore kit contains prevalidated proprietary probes and primers specific to each mutation. While the exact probe sequences are not publicly disclosed by the manufacturer, the kit complies with CE-IVD certification for clinical diagnostics. Data analysis and genotype determination were conducted using CLC Sequence Viewer version 8.0 (Qiagen Bioin-formatics, Aarhus, Denmark), in accordance with the analysis protocol provided in the kit manual. Only female participants with a complete thrombophilia profile covering all six SNPs were included in the final analysis. This methodological information has been expanded in the manuscript to ensure reproducibility and transparency, in response to reviewer feedback.

### 4.5. Statistical Analyses

The statistical analysis compared the distribution of heterozygous and/or homozygous genotypes with the wild-type genotype for each polymorphism. For the *F5* Leiden and *F2* G20210A variants, the dominant inheritance model was applied, while both dominant and codominant models were used for the *MTHFR*.

The Kruskal–Wallis test was employed to compare the median ranks of ordinal (discrete) variables across multiple groups of varying sizes. The Spearman correlation coefficient (ρ) was used to assess the relationship between two ordinal variables within the same group, identifying the direction and strength of either a direct or inverse correlation.

Logistic regression analysis was used to model the relationship between one or more independent variables (xᵢ), which may be categorical or continuous, and a binary (dichotomous) dependent variable (Y). In the case of a single predictor variable, the odds ratio (OR)—a key concept in logistic regression—was calculated to quantify the effect of the predictor on the outcome. For models involving multiple predictors, multiple logistic regression was used to estimate the probability of inclusion of each predictor within one of the outcome categories defined by the dependent variable. The analysis yielded regression coefficients (including the intercept and B coefficients), which represent the individual contribution of each predictor to the model. Categorical variables were compared using the Chi-square test (χ^2^) to assess associations between genotypes and clinical outcomes. A *p*-value < 0.05 was considered statistically significant.

## 5. Conclusions

This study highlights the complex and multifactorial nature of recurrent pregnancy loss (RPL), with a particular focus on the potential contribution of inherited thrombophilic SNPs. Although isolated SNPs in *MTHFR* (C677T and A1298C), *F5* Leiden (G1691A), and *F2* G20210A showed limited individual association with early RPL, our findings suggest that the coexistence of multiple SNPs—especially homozygosity for *MTHFR* C677T in combination with heterozygous *F5* Leiden—may be associated with an increased frequency of pregnancy losses.

These observations are consistent with the hypothesis that cumulative genetic burden, rather than isolated single-gene effects, may contribute to placental vascular dysfunction and impaired gestational progression. In this context, a multigenic screening strategy could potentially offer additional insight compared to single-variant testing, particularly in cases of idiopathic RPL, although this requires further validation.

From a clinical perspective, these findings suggest a potential association that may be relevant for future research into risk stratification in women with unexplained early pregnancy loss. However, no direct clinical implications can be drawn at this stage, and these observations should not be used to guide therapeutic decisions. In addition, the absence of homocysteine and serum folate level assessment limits the interpretation of the functional impact of *MTHFR* polymorphisms, and therefore the biological significance of these associations should be interpreted with caution. Given the study’s limited sample size and the variability reported in the literature, larger, prospective, and ethnically diverse studies are needed to validate these associations and to assess whether tailored interventions based on genetic profiles can improve reproductive outcomes.

## Figures and Tables

**Table 1:** Mutation Co-occurrence—Heterozygous and Homozygous Variants.

SNPs	G1691A (*n* = 9)	G20210A (*n* = 2)	A1298C (*n* = 34)	C677T (*n* = 37)
Heterozygous				
G1691A	–			
G20210A	1 (11.1%)	–		
A1298C	5 (55.6%)	1 (50.0%)	–	
C677T	3 (33.3%)	2 (100%)	20 (58.8%)	–
Homozygous				
G1691A	–			
G20210A	–	–		
A1298C	–	–	–	
C677T	1 (11.1%)	–	1 (2.9%)	–

**Table 2:** Correlation Between Number of Miscarriages and Factor V Leiden Mutation.

G1691A Status	N	Mean Rank	Kruskal–Wallis Test
Heterozygous	9	52.44	Chi-Square = 16.58
Negative	55	29.24	df = 1, *p* = 0.001

**Table 3:** Correlation Between Number of Miscarriages and *F2* G20210A Polymorphism.

G20210A Status	N	Mean Rank	Kruskal–Wallis Test
Heterozygous	2	56.50	Chi-Square = 4.73
Negative	62	32.00	df = 1, *p* = 0.030

**Table 4:** Comparison of Number of Miscarriages Across *MTHFR* A1298C Genotypes (Kruskal–Wallis Test).

A1298C Genotype	N	Mean Rank of Miscarriage Count
Homozygous Mutant (CC)	5	49.40
Heterozygous (AC)	34	28.21
Wild Type (AA)	25	34.96

Kruskal–Wallis Test: χ^2^ = 8.87, df = 2, *p* = 0.012.

**Table 5:** Comparison of Age Across *MTHFR* C677T Genotypes (Kruskal–Wallis Test).

C677T Genotype	N	Mean Rank of Age
Homozygous Mutant (TT)	8	22.19
Heterozygous (CT)	37	30.82
Wild Type (CC)	19	40.11

Kruskal–Wallis Test: χ^2^ = 5.975, df = 2, *p* = 0.050.

**Table 6:** Comparison of Number of Miscarriages Across *MTHFR* C677T Genotypes (Kruskal–Wallis Test).

C677T Genotype	N	Mean Rank of Miscarriage Count
Homozygous Mutant (TT)	8	52.30
Heterozygous (CT)	37	25.86
Wild Type (CC)	19	37.05

Kruskal–Wallis Test: χ^2^ = 20.626, df = 2, *p* = 0.001.

**Table 7:** Prognostic Risk of Four Miscarriages Based on SNPs Combinations.

Mutation Combination	Predicted Probability of 4 Miscarriages
*MTHFR C677T* (homozygous) + *MTHFR A1298C* (heterozygous)	96.2%
*MTHFR C677T* (heterozygous) + *MTHFR A1298C* (heterozygous)	4.0%
*MTHFR C677T* (heterozygous) + *MTHFR A1298C* (heterozygous) + *Factor V Leiden* (heterozygous)	76.3%

**Table 8:** Predictive Probability of Pregnancy Loss by Polymorphism Type.

G1691A	C677T	A1298C	Pregnancy Losses	Observed	Predicted	Pearson Residual	Observed %	Predicted %
negative	negative	heterozygous	3	0	0.045	−2.114	0.0%	5.7%
negative	negative	heterozygous	4	0	0.006	−0.856	0.0%	0.8%
negative	negative	heterozygous	5	0	0.000	0.000	0.0%	0.0%
negative	homozygous	negative	2	3	3.000	0.000	100.0%	100.0%
negative	heterozygous	negative	3	11	1.451	3.407	84.6%	11.2%
negative	heterozygous	negative	4	2	0.154	4.481	15.4%	1.2%
negative	heterozygous	heterozygous	2	11	11.475	−0.222	91.7%	95.6%
negative	heterozygous	heterozygous	3	1	0.194	1.817	8.3%	1.6%
negative	heterozygous	heterozygous	4	0	0.341	−1.847	0.0%	2.8%
negative	homozygous	heterozygous	2	0	0.000	0.000	0.0%	0.0%
negative	homozygous	heterozygous	3	3	3.000	0.000	100.0%	100.0%
negative	homozygous	heterozygous	4	0	0.000	0.000	0.0%	0.0%
heterozygous	negative	negative	4	1	0.952	0.048	100.0%	100.0%
heterozygous	heterozygous	negative	3	1	0.062	3.748	100.0%	100.0%
heterozygous	homozygous	negative	3	0	0.000	0.000	0.0%	0.0%
heterozygous	heterozygous	heterozygous	4	1	0.482	0.748	100.0%	100.0%
homozygous	heterozygous	heterozygous	4	2	1.632	0.672	100.0%	81.6%
homozygous	heterozygous	heterozygous	5	0	0.368	−0.672	0.0%	18.4%
negative	negative	negative	2	0	0.000	0.000	0.0%	0.0%
negative	heterozygous	negative	3	0	0.475	−0.689	0.0%	23.7%
negative	heterozygous	negative	4	1	1.525	−0.424	100.0%	76.3%
negative	homozygous	negative	3	0	0.571	−0.756	0.0%	57.1%
negative	homozygous	negative	4	1	0.429	0.875	100.0%	42.9%
negative	heterozygous	heterozygous	3	1	0.000	0.000	100.0%	100.0%
negative	heterozygous	heterozygous	4	0	0.000	0.000	0.0%	0.0%
negative	heterozygous	heterozygous	5	0	0.000	0.000	0.0%	0.0%
negative	homozygous	heterozygous	3	0	0.500	−0.707	0.0%	50.0%
negative	homozygous	heterozygous	4	1	0.500	0.707	100.0%	50.0%
heterozygous	homozygous	heterozygous	4	1	1.000	0.000	100.0%	100.0%
negative	heterozygous	heterozygous	3	2	2.000	0.000	100.0%	100.0%
negative	heterozygous	heterozygous	4	1	1.000	0.000	100.0%	100.0%
negative	heterozygous	heterozygous	5	0	0.000	0.000	0.0%	0.0%

## Data Availability

The original contributions presented in this study are included in the article. Further inquiries can be directed to the corresponding author.
